# Incorporation of feeding functional group information informs explanatory patterns of long-term population changes in fish assemblages

**DOI:** 10.7717/peerj.11032

**Published:** 2021-03-29

**Authors:** Jason C. Doll, Stephen J. Jacquemin

**Affiliations:** 1 Freshwater Ecology Center, Department of Biology, Francis Marion University, Florence, SC, USA; 2 Agriculture and Water Quality Educational Center, Wright State University—Lake Campus, Celina, OH, USA

**Keywords:** Southern Lake Michigan, Feeding guilds, Bayesian analysis, Hierarchical model

## Abstract

The objective of this study was to evaluate long term trends of fish taxa in southern Lake Michigan while incorporating their functional roles to improve our understanding of ecosystem level changes that have occurred in the system over time. The approach used here highlighted the ease of incorporating ecological mechanisms into population models so researchers can take full advantage of available long-term ecosystem information. Long term studies of fish assemblages can be used to inform changes in community structure resulting from perturbations to aquatic systems and understanding these changes in fish assemblages can be better contextualized by grouping species according to functional groups that are grounded in niche theory. We hypothesized that describing the biological process based on partial pooling of information across functional groups would identify shifts in fish assemblages that coincide with major changes in the ecosystem (e.g., for this study, shifts in zooplankton abundance over time). Herein, we analyzed a long-term Lake Michigan fisheries dataset using a multi-species state space modeling approach within a Bayesian framework. Our results suggested the population growth rates of planktivores and benthic invertivores have been more variable than general invertivores over time and that trends in planktivores can be partially explained by ecosystem changes in zooplankton abundance. Additional work incorporating more ecosystem parameters (e.g., primary production, etc.) should be incorporated into future iterations of this novel modeling concept.

## Introduction

As aquatic habitat losses and alterations have accumulated over the past century, a growing number of fish species have either been extirpated from native areas or gone extinct (Burkhead, 2012). And while these taxonomic losses have occurred at an exceedingly high pace, particularly in freshwater fishes, without long term datasets to characterize these losses it is impossible to truly measure this level of biodiversity change (Jacquemin & Pyron, 2011). Moreover, without incorporating functional information into analytical and management frameworks, it is also impossible to understand the ecosystem level changes that have occurred over time as a result (Hoeinghaus, Winemiller & Birnbaum, 2007). To meet this need, long term studies of fish assemblages have been increasingly used in recent years to inform changes in community structure resulting from alterations to aquatic systems worldwide (Magurran et al., 2010). Grouping species according to functional groups has also improved understanding of how changes have shifted systems at an ecosystem level (Rogers et al., 2014). Grounded in niche theory, functional groupings play an important role in understanding long term change as they help to minimize variation inherent in taxa numbers as well as prioritize ecosystem-level management and conservation strategies (Pyron et al., 2011).

A potential limitation of grouping taxa into functional groups by ecosystem role, however, could be the trade of taxonomic information for functional grouping categories, providing alternative assumptions on the emergent data (Hoeinghaus, Winemiller & Birnbaum, 2007; Jacquemin & Pyron, 2011; Pyron et al., 2011). In this context, two datasets would depict the species level taxonomic data and the combined taxa by functional roles in the community, respectively. The former assumes that species are independent, exhibiting “no pooling” of information in the dataset, whereas the later assumes exchangeability across species within the functional role (i.e., trends in two species within the same functional role follow the same trajectory), exhibiting “complete pooling”. Complete pooling of data into functional roles provides useful information to better understand an ecosystem, however, it could also result in lost information as taxonomic information may inform different aspects of ecosystem change and vice versa (Jacquemin & Pyron, 2011).

The fish community in Lake Michigan provides a unique opportunity to evaluate how modeling known functional roles of species over a long period of time can influence inference because of the documented long-term ecosystem changes (Mida et al., 2010) coupled with availability of long term data (Lauer, Allen & McComish, 2004). Specifically, the factors that structure the fish community of Lake Michigan primarily relate to food web changes; including stocking of top predators (e.g., Chinook Salmon *Oncorhynchus tshawytscha* and Lake Trout *Salvelinus namaycush*) and their consumption of prey fish (Tsehaye et al., 2014), proliferation of invasive species, as the Round Goby *Neogobius malenostomus*, which impact trophically similar species (Mills et al., 1993; Lauer, Allen & McComish, 2004; Hondorp, Pothoven & Brandt, 2005; Nalepa, Fanslow & Lang, 2009), and changes in lake productivity (i.e., related to reduced phosphorus load; Bunnell, Madenjian & Claramunt, 2006). Notably, amidst the ample literature available about fish assemblages of Lake Michigan (Rand et al., 1995; Bunnell, Madenjian & Claramunt, 2006; Bhagat & Ruetz, 2011; Tsehaye et al., 2014), there is still a paucity of relevant information about long term changes of fisheries functional groups in the southern part of Lake Michigan.

A key concept that underlies the challenges in understanding long-term community dynamics is that species-specific trends often contradict with ecological roles in the ecosystem (Lauer, Allen & McComish, 2004). This concept is highlighted within the Lake Michigan fish community where a reduction in Mottled Sculpin and Johnny Darters coincided with an increase in Round Gobies, three trophically similar species that do not exhibit the same abundance trends (Lauer, Allen & McComish, 2004). Thus, treating each species independently ignores the similarities in abundance trends between Mottled Sculpin and Johnny Darter and grouping all species together in the same functional group masks the different trajectories of all three species. In this case, taking an approach at either extreme (i.e., no pooling or complete pooling) can remove valuable information about species diversity. In contrast, an intermediate approach that shares information across species but also provides species specific trends could be more appropriate. The approach outlined in this study is to incorporate functional traits from life history information into taxonomic analyses, preserving taxonomic information while also considering known ecological niche based roles in the biological process to improve long term explanations of fish assemblage change (i.e., statistical “partial pooling” with random effects).

The specific objective of the current study was to evaluate long term trends of taxa while incorporating their functional role in the southern Lake Michigan ecosystem. To this end, the long-term changes in the near-shore fish community of southern Lake Michigan were described using a multi-species state-space model of population growth rates that integrates functional feeding groups. Based on the aforementioned, the approach assumes partial exchangeability across species within each group by incorporating a random effect at the biological process level (i.e., statistical “partial pooling” with random effects). We hypothesize that describing the biological process based on partial pooling of information across functional groups might identify shifts in the fish assemblage that coincide with some of the already documented major changes in the food web (e.g., zooplankton abundance).

## Methods

### Overview of the experimental program

**Figure 1:**
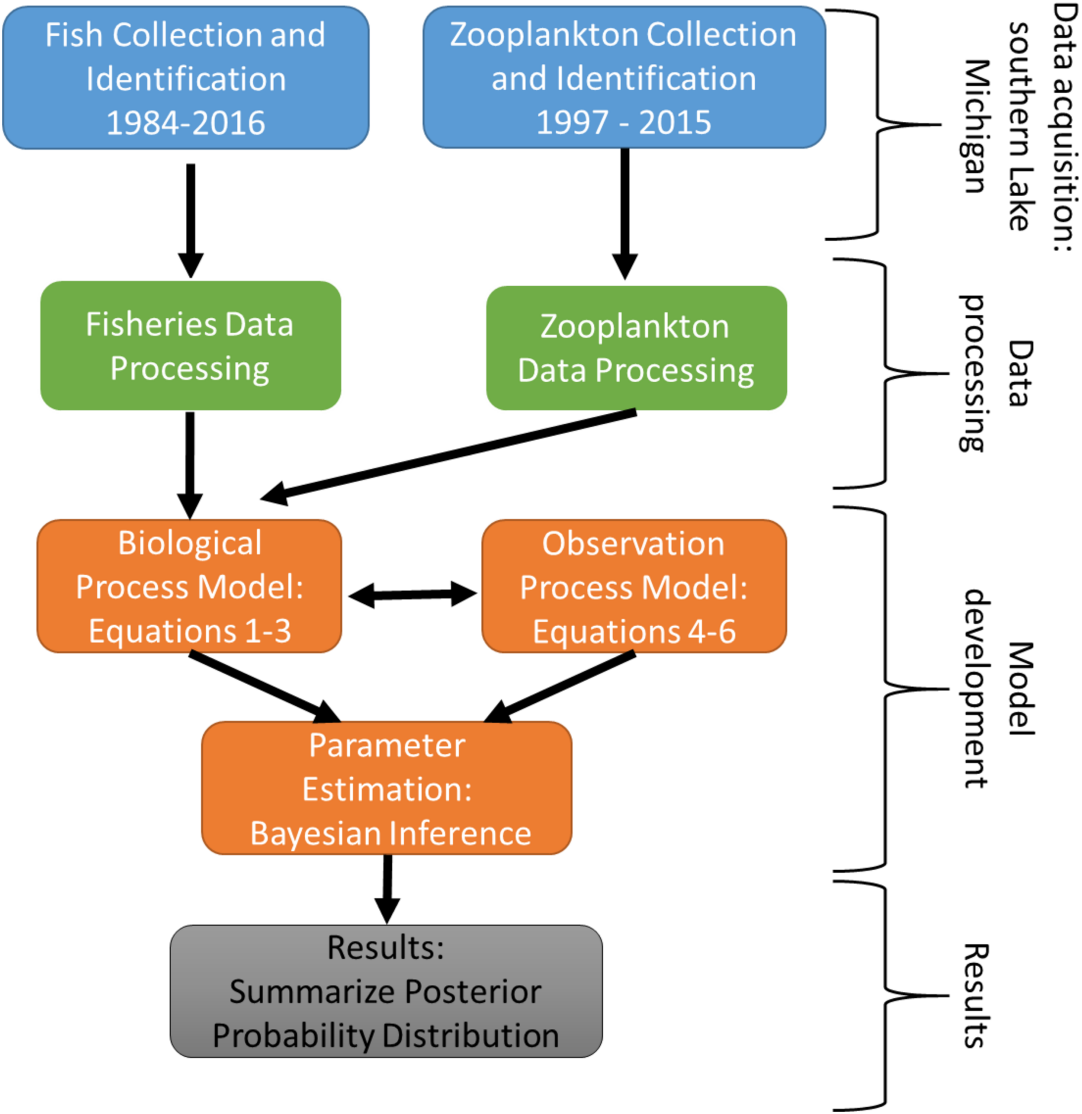
Schematic diagram of the experimental program in southern Lake Michigan that occurred between 1984 and 2016. Blue boxes represent data acquisition (fisheries survey and zooplankton survey), green boxes represent data acquisition and processing, orange boxes represent statistical modeling, and gray box represents summary of results.

**Figure 2:**
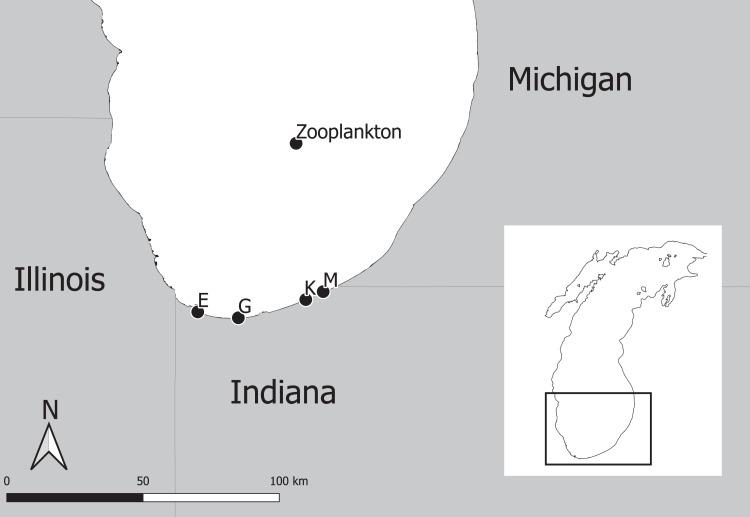
Map of four fish sampling sites (East Chicago (E), Burns Harbor (G), Kintzele Ditch (K), and Michigan City (M)) and one zooplankton sample site in southern Lake Michigan.

The experimental program in southern Lake Michigan occurred between 1984 and 2016 and integrated a robust fisheries survey between 1984 and 2016, zooplankton survey between 1997 and 2015, and statistical modeling (Fig. 1). The objective of this study was to evaluate long term trends of fish taxa in southern Lake Michigan while incorporating their functional role in the ecosystem. A map of the four fish sampling sites, and one zooplankton sample site is depicted in Fig. 2. Fish sampling was conducted using a semi-balloon bottom trawl. Sites were not consistent across the study period as additional sites were added to expand the long-term monitoring program. See “Study area and data collection” for more detail on sites. All captured fish were identified to species level and counted during fisheries data processing. Fish species were grouped according to both functional feeding guilds (Trautman, 1981; Poff & Allan, 1995; Hondorp, Pothoven & Brandt, 2005; Truemper et al., 2006; Happel et al., 2015). Zooplankton data were acquired from a U.S. EPA database. Zooplankton were sampled at a fixed site using vertical tows of a 153-μm mesh net. Long-term trends in the fish assemblage were described using a multi-species exponential population growth model in a state space framework (Kéry & Schaub, 2012; Doll et al., 2020). This modeling approach separates the biological process model from the observation process model to separate variation from the two sources. The biological process model was parameterized to incorporate shared information across fish species within a functional feeding group by incorporating a random effects term (see Eqs. (2) and (3) below). The biological process model is further parameterized to explore the relationship between the population growth rate and zooplankton abundance. Parameters of the model are estimated using Bayesian inference. Results and conclusions were made using the full joint posterior probability distribution of model parameters.

### Study area and data collection

Southern Lake Michigan is generally flat, less than 20 m deep and sandy (Janssen, Berg & Lozano, 2005). Fish were sampled at up to four sites between 1984 and 2016 (Fig. 2) using a semi-balloon bottom trawl with 4.9 m headrope, 5.8 m footrope, 38 mm stretch mesh body with a 32 mm stretch mesh cod end lined with 13 mm stretch mesh liner. Two sites (M and K) were sampled between years 1984 and 1988, three sites (M, K, and G) were sampled between 1989 and 2009, and four sites (M, K, G, and E) were sampled between 2010 and 2016. Sampling at each site occurred twice a month in June, July, and August between 1984 and 2009; and sampling occurred once a month at each site in August between 2010 and 2016. Trawling occurred between sunset and midnight along the 5 m depth contour. A total of 1 h of trawling was conducted at each sampling event. Total effort increased throughout the sampling program and ranged from 12 to 34 h. All captured fish were identified to species level and counted. Catch per unit effort (CPUE) represented total catch (in number) per hour of trawl sampling. Sampling protocol was approved by the Ball State University Institutional Animal Care and Use Committee (IRB number: 870492-1).

### Zooplankton data

Zooplankton data were extracted from the Great Lakes National Program Office (GLNPO) database on November 12, 2019 (United States Environmental Protection Agency Great Lakes National Program Office, 2019). In particular, the seasonal variation and spatial variability were reduced using only the summer samples and from site MI 11 (42.38333, −87.00000, depth = 128 m; Fig. 2). Zooplankton were sampled with a 153-μm mesh net using a vertical tow starting at 100 m deep. Samples were narcotized with soda water and preserved with sucrose formalin. Each sample was split using a Folsom plankton splitter until approximately 200–400 animals were present in a split. Two splits were counted and identified with a stereoscope. Taxonomy followed Balcer, Korda & Dodson (1984), Hudson et al. (1998), Brooks (1957), Evans (1985), and Rivier (1998). Dry weights were estimated using a length-weigh regression following United States Environmental Protection Agency (2013). Annual mean dry weights for Calanoid copepod adults, Cyclopoid copepod adults, *Daphnia*, non-daphnid herbivorous cladocerans, and predatory cladocerans were summed for an annual total biomass estimate. Zooplankton data were standardized to z-scores to improve model fitting efficiency. See United States Environmental Protection Agency Great Lakes National Program Office (2019) for complete description of data and protocols used by the Environmental Protection Agency.

### Functional groups

Species were grouped according to both functional feeding guilds described by Poff & Allan (1995) using basic life history information outlined in Trautman (1981) and several Lake Michigan specific dietary studies of taxa (Hondorp, Pothoven & Brandt, 2005; Truemper et al., 2006; Happel et al., 2015). Functional feeding group classifications for observed species with descriptive statistics is shown in Table 1. Notably, the lack of diversity in the assemblage of Lake Michigan permitted to assign the feeding guilds consistent with predominant age structures. Overall, the emerging dataset included planktivore, general invertivore, and benthic invertivore guilds (Table 1).

**Table 1:** Functional feeding group classifications for observed species with average (standard deviation (sd)), first quartile, third quartile, and maximum annual catch per unit effort.

Species	Functional Group	Mean (sd)	Q1	Q3	Max
White Sucker (*C*. *commersonii*)	Benthic invertivore	0.7 (4.4)	0.0	0.0	91.0
Longnose Sucker (*Catostomus catostomus*)	Benthic invertivore	1.0 (2.2)	0.0	1.0	17.0
Johnny Darter (*Etheostoma nigrum*)	Benthic invertivore	3.4 (10.6)	0.0	2.0	114.0
Round Goby (*Neogobius melanostomus*)	Benthic invertivore	44.4 (132.2)	0.0	5.75	1285.0
Yellow Perch (*Perca flavescens*)	General invertivore	460.9 (845.1)	28.0	471.3	6269.0
Spottail Shiner (*Notropis hudsonius*)	General invertivore	529.4 (691.2)	122.5	619.0	4270.0
Troutperch (*Percopsis omiscomaycus*)	General invertivore	8.3 (29.1)	0.0	3.0	258.0
Rainbow Smelt (*Osmerus mordax*)	Planktivore	26.8 (438.3)	0.0	0.0	9397.0
Bloater (*Coregonus hoyi*)	Planktivore	37.4 (321.0)	0.0	0.0	5497.0
Alewife (*Alosa pseudoharengus*)	Planktivore	83.5 (124.1)	10.0	103.5	831.0

### State space model

#### Biological process model

Temporal trends of all species were evaluated using a state space modeling approach (Kéry & Schaub, 2012; Doll et al., 2020). State space models have been widely used in fisheries and ecology to better understand long-term changes in population structure. Specific applications include stock assessments (Nielsen & Berg, 2014; Aeberhard, Flemming & Nielsen, 2018), animal movement and migration (Jonsen, Myers & James, 2006; Patterson et al., 2008), identify metapopulation structure (Ward et al., 2010), and understanding foraging tactics of gray seals *Halichoerus grypus* (Breed et al., 2009). The base model is similar to the model used in Doll et al. (2020). The species specific biological process model (i.e., population dynamics model) was assumed to follow the exponential growth model:

(1) }{}{N_{t + 1}} = {N_t}{{\rm \lambda}_t}

where *N*_*t*+1_ represents the population size during time *t*+1, *N*_*t*_ represents the population size at time *t* and }{}{\lambda _t} represents the population growth rate at time *t* and *t* ranges from 1 to the total number of years where data were collected. Equation (1) represents the true but unknown process of the population for species *s* (i.e., no observation error). The model specification assumes the population growth rate parameter of species within the same functional feeding group are similar where species within the same functional feeding group share the same mean population growth rate, }{}{\bar {\rm \lambda} _{f,s,t}} with a random effect for species within the functional group:

(2) }{}{N_{s,t + 1}} = {N_{s,t}}{{\rm \lambda}_{f,s,t}}

(3) \matrix{ {{\rm Years} = 1984\!-\!1996} \cr {{\rm Years} = 1997\!-\!2015} \cr } \; \; \; \; \; \matrix{ {{{\rm \lambda} _{f,s,t}} \sim {\rm Normal}({{{\bar {\rm \lambda}}_f},{{\rm \sigma} _{{\rm \lambda} ,f}}})} \cr {{{\rm \lambda} _{f,s,t}} \sim {\rm Normal} ({{{\rm {\alpha}} _f} + {{\rm \beta} _f}*{X_t},{{\rm \sigma} _{{\rm \lambda},f}}})} \cr }

where }{}{\bar \lambda _f} is the mean population growth rate for functional groups *f*, }{}{{\rm \sigma} _{{\rm \lambda} ,f}} is the standard deviation of the population growth rate for functional feeding group *f*, }{}{{\rm \alpha} _f} is the intercept when describing the population growth rate as a function of zooplankton biomass for functional feeding group *f*, }{}{{\rm \beta} _f} is the slope of the effect of zooplankton biomass on the population growth rate for functional feeding group *f*, }{}{X_t} is the observed zooplankton biomass in year *t*, and }{}{{\rm \lambda} _{f,s,t}} is species specific mean population growth rate for each year *t*. Zooplankton data are not available for the full time period of fisheries data, therefore, we modeled the biological process in two stages; years 1984–1996 with an overall mean population growth rate and 1997–2015 where the annual population growth rate for each functional feeding group varied by observed zooplankton biomass (Eq. (3)). The initial population, *N*_*s*,*1*_, is not defined, therefore we are estimating it as a separate parameter using a non-informative prior probability distribution (Table 2).

**Table 2:** Prior probability distributions used in the state-space population model.

Parameter		Equation	Prior distribution
}{}{{\rm \sigma}_{{\rm \lambda} ,f}}	Standard deviation for process error	3	Cauchy (0, 2)^+^
}{}{\bar {\rm \lambda} _f}	Mean population growth rate	3	Normal (0, 2)
N_*s*,1_	Initial abundance (on log scale)	3	Normal (0, 2)
}{}{{\rm \alpha} _f}	Intercept parameter	3	Normal (0, 2)
}{}{{\rm \beta} _f}	Slope parameter	3	Normal (0, 2)
}{}{{\epsilon}_{i, s}}	Individual random effect for overdispersion	5	Normal (0, }{}{{\rm \sigma} _S})
γ_*S*,*site*_	Site random effect	5	Normal (0, σ_*S*,*site*_)
}{}{{\rm \sigma} _S}	Standard deviation for individual random effect		Cauchy (0, 2)^+^
}{}{{\rm \sigma} _{S,site}}	Standard deviation for site random effect of transect survey		Cauchy (0, 2)^+^

**Note:**

+ The positive values of the Cauchy distribution resulting in a half-Cauchy prior probability distribution. The half-Cauchy distribution was used as a prior probability distribution for each standard deviation parameter following the recommendations of Gelman (2006). The normal distribution was selected for other parameters as a default prior probability distribution to have minimal influence on the posterior probability distribution.

#### Observation process model

A second set of equations are used to link the population dynamics model to the observation dataset.

(4) }{}{{y}_{i,s}} \sim {\rm Poisson}\left( {{{\rm \mu} _{i,s}}} \right)

where *y_i,s_* is the observed counts of species *s* during the survey and }{}{{\rm \mu} _{i,s}} is the mean parameter of the survey for observation *i*. The mean and variance of the Poisson distribution are equal and defined as:

(5) }{}{\rm {\open E}}\left( {{{y}_{i,s}}} \right) = Var\left( {{{y}_{i,s}}} \right) = {{\rm \mu} _{i,s}}

The log link was used to model dependencies in the mean as a function of covariates. Further, because count data can be overdispersed, we incorporated a normal random effect in the linear predictors following Kéry & Schaub (2012). The standard Poisson and Negative binomial distribution were also considered to account for overdispersion but they were deemed inappropriate based on model diagnostics and thus the details are not included here. The model is described as a Poisson-lognormal model with a random effect for site and an overdispersion parameter.

(6) }{}{{\rm \mu} _{i,s}} = {\rm exp}\left( {{N_{s,t}} + {{\rm \gamma} _{s,site}} + {{\rm \varepsilon} _{i,s}}} \right)

where }{}{{\rm \gamma} _{s,site}} is a random effect term for species *s* at sampling site, and }{}{{\rm \varepsilon} _{i,s}} is a random effect for the individual to capture overdispersion.

Bayesian inference was used to fit the model (Doll & Jacquemin, 2018) in the programing languages R 3.6.1 (R Core Team, 2019), Stan (Stan Development Team, 2018a), and rstan 2.17.3 (Stan Development Team, 2018b). All parameters were given non-informative priors (Table 2). Three concurrent Markov Chain Monte Carlo (MCMC) chains were used. Each chain consisted of 15,000 total iterations (split between the three chains). The first 2,000 steps of each chain were discarded for a total of 9,000 saved steps. The split \hat R and visual inspection of traceplots were used to assess convergence. The chains have converged when the split \hat R is close to one. Values less than 1.1 suggests the MCMC chains have converged. Parameters are summarized by the median and posterior 95% credible intervals (CRI).

## Results

**Figure 3:**
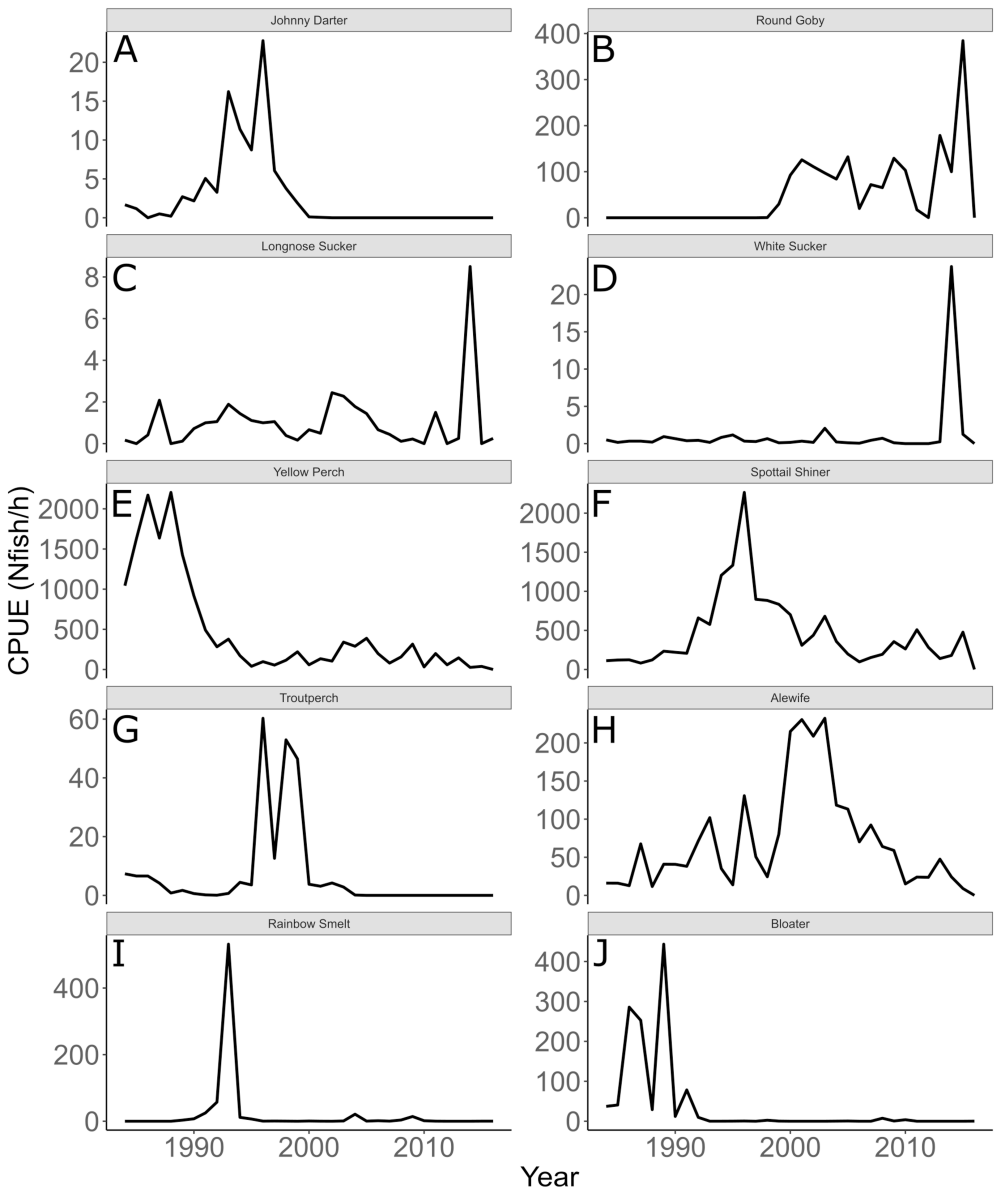
Annual mean trawl catch per unit effort. Annual mean trawl catch per unit effort (CPUE) expressed as *N*_fish_/*h* of species sampled in southern Lake Michigan between 1984 and 2016. Note different *y*-axis scale for each species. (A) Johnny Darter, (B) Round Goby, (C) Longnose Sucker, (D) White Sucker, (E) Yellow Perch, (F) Spottail Shiner, (G) Troutperch, (H) Alewife, (I) Rainbow Smelt, (J) Bloater.

**Figure 4:**
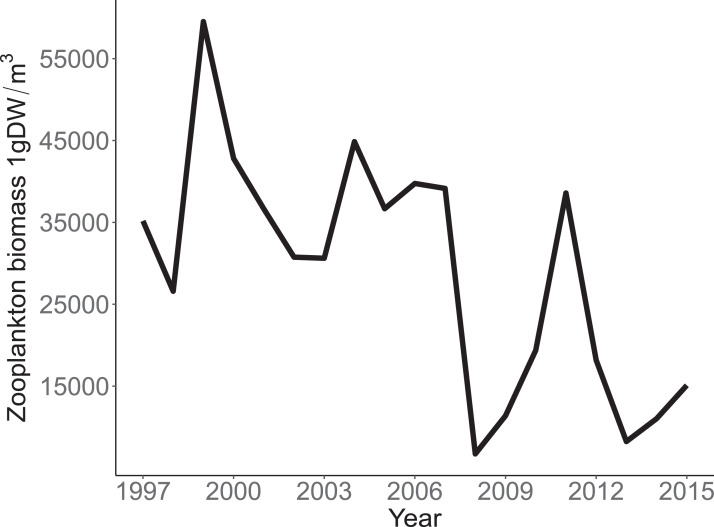
Annual total Zooplankton biomass sampled at site MI11 in southern Lake Michigan by the USEPA between 1997 and 2015. The solid line represents the total dry weights of Calanoid copepod adults, Cyclopoid copepod adults, Daphnia, non-daphnid herbivorous cladocerans, and predatory cladocerans.

Annual trends in CPUE of each species are depicted in Fig. 3. Temporal trends in CPUE of individual species were highly variable (Fig. 3). Johnny Darter, Yellow Perch, Spottail Shiner *Notropis hudsonius*, Troutperch *Percopsis omiscomaycus*, Rainbow Smelt *Osmerus mordax*, and Bloater *Coregonus hoyi* exhibited peak abundance in the 1980’s and 1990’s whereas Alewife exhibited peak abundance in the 2000’s with Round Goby, Longnose Sucker *Catostomus catostomus*, and White Sucker *Catostomus commersonii* abundance peaking in 2010’s. Spottail Shiners were the most abundant species overall followed by Yellow Perch, Alewife, Round Goby, Bloater, and Rainbow Smelt (Table 1). The remaining four species averaged less than 10 fish/h. Trends in zooplankton abundance are depicted in Fig. 4. Over the past 20 years, zooplankton abundance followed a decreasing trend between 1997 and 2015 (Fig. 4) with peak abundance within the time series as 59,522 μgDW/m^3^ in 1999 and the lowest abundance as 6,720 μgDW/m^3^ in 2008. Overall, average abundance of zooplankton was 29,006 μgDW/m^3^ (SD = 14,559) per year.

**Figure 5:**
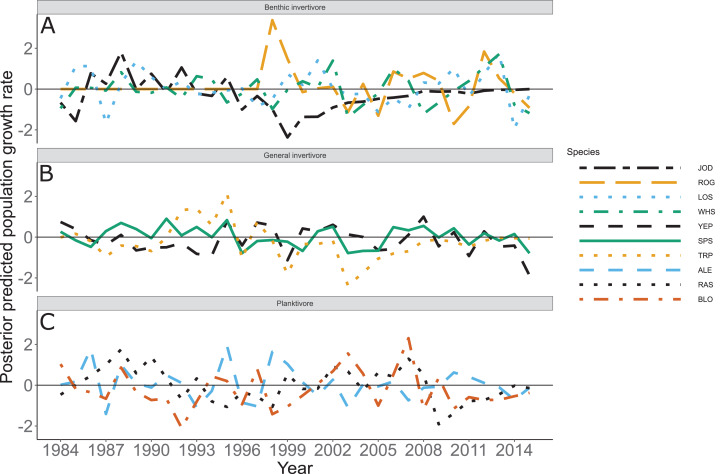
Posterior predicted population growth rate by species and year. Species separated by functional feeding groups; (A) Benthic invertivore (Johnny Darter (JOD), Round Goby (ROG), Longnose Sucker (LOS), and White Sucker (WHS)); (B) General invertivore (Yellow Perch (YEP), Spottain Shiner (SPS), and Troutperch (TRP)); (C) Planktivore (Alewife (ALE), Rainbow Smelt (RAS), and Bloater (BLO)). Lines represent medians of the posterior probability distribution and horizontal line intercepting *y*-axis at 0 is for reference only.

**Figure 6:**
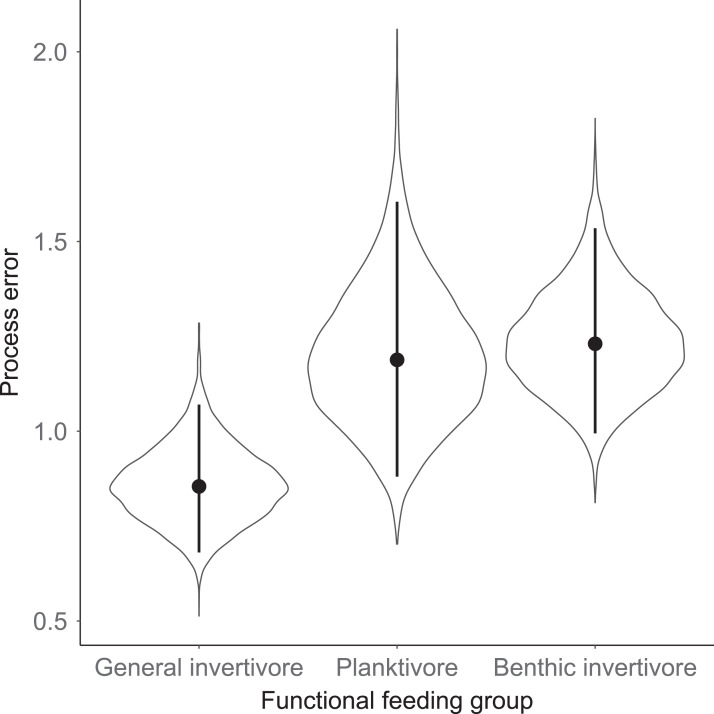
Standard deviation of the population growth rate parameter for each functional group. Solid circles represent medians of the posterior distribution, solid vertical bars represent bounds of the 95% Bayesian Credible Interval, and violin plots represent a mirrored density plot of the full posterior probability distribution.

**Figure 7:**
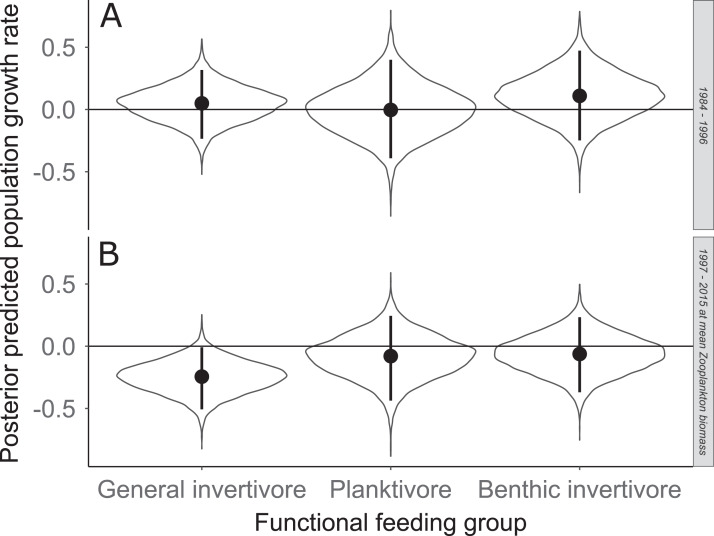
Posterior predicted population growth rate for each functional feeding groups prior to zooplankton data (1984–1996; A) and during (1997–2015; B). Estimates with zooplankton represent expected population growth rate at mean zooplankton biomass. Solid circles represent medians of the posterior distribution, solid vertical bars represent bounds of the 95% Bayesian Credible Interval, violin plots represent a mirrored density plot of the full posterior probability distribution, and solid horizontal bars are reference bars for 0.

**Figure 8:**
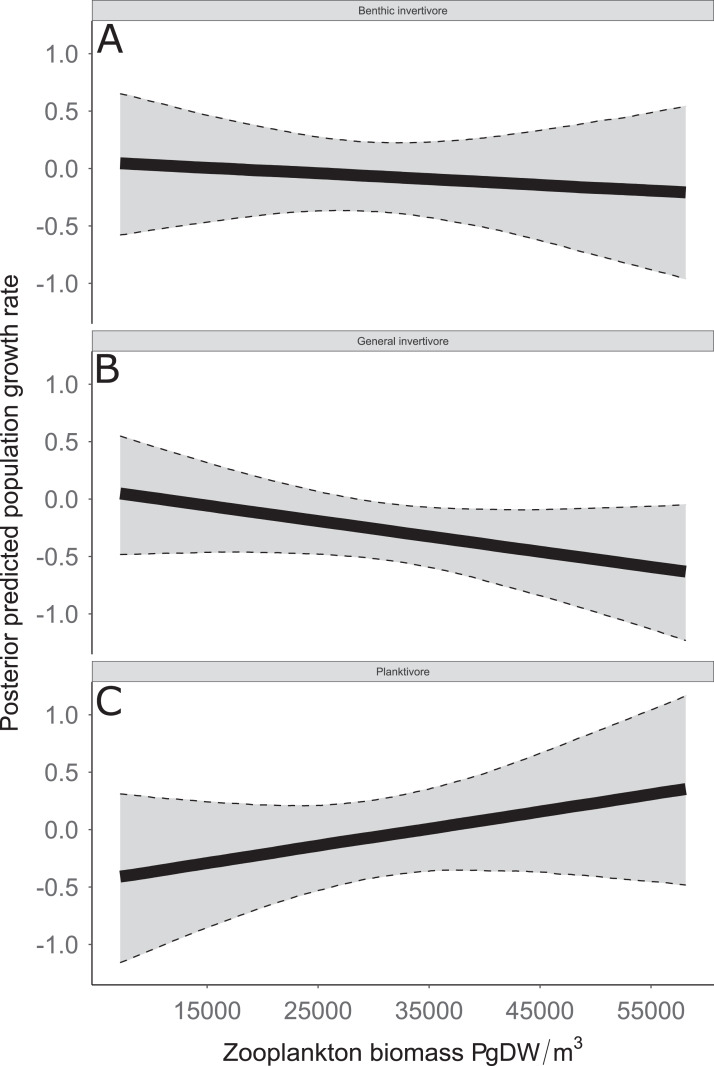
Posterior predicted population growth rate as a function of zooplankton biomass (μg dry weight (DW)/m^3^). Functional feeding groups separated by benthic invertivore (A), general invertivore (B), and planktivore (C). Solid lines represent median of the posterior predictive values and bounds of the gray ribbon represent upper and lower 95% Bayesian posterior credible intervals.

Species specific population growth rate over time are shown in Fig. 5 and categorized by functional feeding group. Yearly population growth rate was variable across species and functional groups (Fig. 5). Variation in yearly population growth rate for each functional feeding group is summarized as processes error in Fig. 6. The general invertivores were more stable with the annual population growth rate fluctuating closely around 0 with the exception of Troutperch (TRP). In contrast, benthic invertivores and planktivores exhibited much greater variability. This variability is reflected in the standard deviation of the functional feeding group population growth rate (i.e., process error; Fig. 6) where the standard deviation of planktivores and benthic invertivores were greater than general invertivores (Fig. 6). The functional feeding group mean population growth rate during the period before zooplankton data and the period with zooplankton data are depicted in Fig. 7. Overall mean population growth rate between 1984 and 1996 suggests a stable population for each functional feeding group (Fig. 7A). In contrast, the expected population growth rate at a mean zooplankton biomass for each functional feeding group were generally negative (Fig. 7B). Figure 8 depicts the trend between zooplankton biomass and the population growth rate for each functional feeding group. The population growth rate of general invertivores was negatively related to zooplankton biomass (median slope = −0.19; 95% CRI [−0.47 to 0.09]), whereas to the population growth rate for planktivores was positively related to zooplankton biomass (median slope = 0.22; 95% CRI [−0.19 to 0.62]), but no strong relationship was observed between the population growth rate of benthic invertivores and zooplankton biomass (median slope = −0.07; 95% CRI [−0.43 to 0.29]). This relationship suggests general invertivores tended to have a stable population (mean population growth rate approximately 0) with respect to zooplankton biomass during years with the lowest zooplankton biomass and a declining population with high zooplankton biomass (Fig. 8). In contrast, the population growth rate of planktivores changed from a declining annual rate at the lowest zooplankton biomass to an increasing annual rate at the highest zooplankton biomass (Fig. 8). Finally, the benthic invertivores exhibit a stable population at all levels of zooplankton biomass (Fig. 8).

## Discussion

The analysis used here demonstrates the ease of which ecological relationships, such as functional level traits, can be combined with long term taxonomic datasets to draw inference at multiple levels without losing important taxonomic or functional resolution. Results of this research also contribute to a better understanding of long term taxonomic and functional changes in the southern Lake Michigan ecosystem. Community surveys documenting long term changes in Lake Michigan have identified a reduction in Alewives, increases in Rainbow Smelt and Yellow Perch, and no long-term changes in Spottail Shiner and Trout-Perch (Jude & Tesar, 1985). A more recent study documented similar species level changes (Bunnell, Madenjian & Claramunt, 2006). For example, Bunnell, Madenjian & Claramunt (2006) described the fish community in the 1970’s as being dominated by Alewife with a shift in the 1980’s to low abundance and increased native fish abundance (e.g., Burbot *Lota lota*, Deepwater Sculpin *Myoxocephalus thompsonii*, and Yellow Perch).

The analysis presented here is largely consistent with these observations but incorporated an ecological concept that links many species at the functional level. For example, see Fig. 3 for trends in raw data. Here we documented taxonomic and functional changes of the near-shore fish community over a 30-year period (Figs. 3 and 7). Specifically, planktivore and benthic invertivore populations were more variable when compared with general invertivores (see Fig. 6). This is likely a function of exotic species exhibiting contrasting trends as general invertivores only included native species whereas planktivores and benthic invertivores included Alewife and Round Goby, respectively. The comparison of native vs non-native species has important implications for refilling of functional niche spaces as native species are often outcompeted. The analysis was extended by incorporating one potential mechanism behind these changes through the addition of zooplankton biomass as an explanatory variable. Specific to zooplankton, we documented a positive relationship with planktivores, no relationship with benthic invertivores, and negative relationships with general invertivores (see Fig. 8). The interesting trend with general invertivores likely reflected that zooplankton was not the primary forage for all general invertivores. Overall, however, the taxonomic changes translated into predictable patterns of functional feeding groups that were partially related to changes in zooplankton density. It should be noted that the fish and zooplankton data were collected at spatially distinct locations and the distance between the two could confound our results. However, we believed the directional relationship was supported by the data and knowledge of the system. For example, a spatial comparison of zooplankton nearshore vs offshore Lake Michigan found no significant difference in biomass and all sites across the depth gradient exhibited a similar decline since the 1970’s (Pothoven & Fahnenstiel, 2015).

Coinciding with these changes in the population growth rate of functional feeding groups in southern Lake Michigan are a series of major perturbations in phytoplankton, zooplankton, invasive species, and nutrient levels. For example, observed annual trends in benthic invertivore species include the invasive Round Goby that was first observed in 1993 (Charlebois et al., 1997). The invasion of Round Goby has been linked to the reduction of other benthic invertivores such as Johnny Darters (Lauer, Allen & McComish, 2004), likely leading to the increased uncertainty in benthic invertivores. Additionally, chlorophyl-α and total phosphorus in Lake Michigan have decreased (Pothoven & Fahnenstiel, 2013) and the water has become clearer since 1998 (Yousef et al., 2017). The increased water clarity coincides with a decrease in zooplankton, thus, it would be expected that feeding groups that rely on zooplankton would follow the same trend. However, the population growth rate of general invertivores in this study is negatively related to zooplankton abundance (see Fig. 8). This would be counterintuitive given strict dietary requirements, however, general invertivores do not rely solely on zooplankton as their primary diet source. Further, one species of general invertivores, Yellow Perch, exhibit euryphagous characteristics and shift their diet throughout their life. Specifically, Yellow Perch transition from a diet primarily consisting of zooplankton to a diet primarily of amphipods, isopods, chironomids, and fish when they reach approximately 120 mm (Turschak et al., 2019). Further, Yellow Perch are not fully recruited to the sampling gear used in this study until they are age-2 (∼125 mm; Forsythe, Doll & Lauer, 2012). Thus, there is likely a lurking variable connecting observed trends in Yellow Perch (and general invertivores) with trends in zooplankton abundance. A potential explanation is the negative relationship between Alewife abundance and Yellow Perch recruitment (Forsythe, Doll & Lauer, 2012). Planktivorous species such as Alewife, Rainbow Smelt, and Bloater, were expected to be positively related to zooplankton given their dietary habits and indeed our observations support this. Thus, the increase in planktivorous fish as a function of zooplankton abundance could explain the negative relationship between general invertivores and zooplankton abundance.

Although our results were consistent with many other observations in Lake Michigan, caution must be taken when trying to extrapolate our results to the greater Lake Michigan ecosystem dynamics. There are many complex interactions that were not included in our model. For example, many fish species in our dataset are not influenced by total zooplankton abundance but rather, abundance of specific size of zooplankton (Bremigan, Dettmers & Mahan, 2003). Larval Yellow Perch, for example, feed predominantly on small copepods and small copepods have declined in southern Lake Michigan (Bremigan, Dettmers & Mahan, 2003). It is important to note that there are additional parameters, such as benthic macroinvertebrates, primary production, and nutrient loading that have also been hypothesized to be related to changes in fish communities (Colby et al., 1972; Bunnell, Madenjian & Claramunt, 2006; Jeppesen et al., 2007; Johannsson et al., 2000), that could also be included into a model such as described above. These parameters were not included due a lack of consistent long-term data describing primary production, nutrient loading, and other macroinvertebrates. Nevertheless, our results remain useful by providing insight at multiple levels of taxonomic and functional groups. Additional insight could be gained by increasing the complexity of interactions across species with the same functional group and adding additional biotic and abiotic covariates that are hypothesized to be important.

## Conclusions

The current study evaluated long term trends of taxa while incorporating their functional role in the southern Lake Michigan ecosystem. A multi-species state-space model of population growth rates integrating functional feeding groups was used to describe long-term changes in the near-shore fish community of southern Lake Michigan used to demonstrate the usefulness of building ecological realism in models describing species trends. Overall, the results of the presently reported study addressed an important topic in assemblage literature related to combing or separating datasets in a gradient of pooling approaches. Such improved knowledge demonstrated how a partial pooling approach (i.e., building a model that is structured in a hierarchical framework to share information across groups) can be taken to incorporate functional traits into a taxonomic dataset. To supplement existing information, future studies of long-term changes using null models should be investigated.

## Supplemental Information

10.7717/peerj.11032/supp-1 Supplemental Information 1 Model code. Code for performing the hierarchical model.

10.7717/peerj.11032/supp-2 Supplemental Information 2 Fish species total catch. Number of fish sampled by site, month, and location.

10.7717/peerj.11032/supp-3 Supplemental Information 3 Zooplankton data. Total zooplankton abundance by year.

10.7717/peerj.11032/supp-4 Supplemental Information 4 R code for running analysis. Code for importing, organizing, and running analysis.

## Additional Information and Declarations

### Data Availability

The following information was supplied regarding data availability: Code and data are available in the Supplemental Files.
